# The mannose 6‐phosphate/insulin‐like growth factor 2 receptor mediates plasminogen‐induced efferocytosis

**DOI:** 10.1002/JLB.1AB0417-160RR

**Published:** 2019-01-18

**Authors:** Anna Ohradanova‐Repic, Christian Machacek, Clemens Donner, Vanessa Mühlgrabner, Eva Petrovčíková, Alexandra Zahradníková, Kristína Vičíková, Václav Hořejší, Hannes Stockinger, Vladimir Leksa

**Affiliations:** ^1^ Molecular Immunology Unit Institute for Hygiene and Applied Immunology Centre for Pathophysiology Infectiology & Immunology Medical University of Vienna Vienna Austria; ^2^ Laboratory of Molecular Immunology Institute of Molecular Biology Slovak Academy of Sciences Bratislava Slovak Republic; ^3^ Department of Biochemistry Faculty of Natural Sciences Comenius University Bratislava Slovak Republic; ^4^ Centre of Biosciences Slovak Academy of Sciences Bratislava Slovak Republic; ^5^ Institute of Molecular Genetics of the Academy of Sciences of the Czech Republic Prague Czech Republic; ^6^ Institute of Experimental Endocrinology Biomedical Research Center Slovak Academy of Sciences Bratislava Slovakia

**Keywords:** M6P/IGF2R, efferocytosis, tissue homeostasis, macrophages, plasminogen

## Abstract

The plasminogen system is harnessed in a wide variety of physiological processes, such as fibrinolysis, cell migration, or efferocytosis; and accordingly, it is essential upon inflammation, tissue remodeling, wound healing, and for homeostatic maintenance in general. Previously, we identified a plasminogen receptor in the mannose 6‐phosphate/insulin‐like growth factor 2 receptor (M6P/IGF2R, CD222). Here, we demonstrate by means of genetic knockdown, knockout, and rescue approaches combined with functional studies that M6P/IGF2R is up‐regulated on the surface of macrophages, recognizes plasminogen exposed on the surface of apoptotic cells, and mediates plasminogen‐induced efferocytosis. The level of uptake of plasminogen‐coated apoptotic cells inversely correlates with the TNF‐α production by phagocytes indicating tissue clearance without inflammation by this mechanism. Our results reveal an up‐to‐now undetermined function of M6P/IGF2R in clearance of apoptotic cells, which is crucial for tissue homeostasis.

**Abbreviations**
AC: apoptotic cells
AF: Alexa Fluor
M6P/IGF2R: mannose 6‐phosphate/insulin‐like growth factor 2 receptor
M‐CSF: macrophage colony‐stimulating factor
Plg: plasminogen
RNAi: RNA interference
shRNA: short hairpin RNA
SSP: staurosporine
TA: tranexamic acid
uPA: urokinase‐type plasminogen activator
uPAR: urokinase‐type plasminogen activator receptor
ZFN: zinc finger nuclease

## 1 INTRODUCTION

The plasminogen activation system, one of the major blood plasma proteolytic pathways, is best known for its role in dissolution of fibrin clots when they are no longer needed. However, the active serine protease plasmin, a central player of the system, participates in many other physiological as well as pathological processes. Activation of plasminogen (Plg), a zymogen of plasmin, must therefore be tightly balanced. On the one hand, rampant plasmin activity contributes to tumor cell invasion,^1^ on the other hand, plasmin deficiency may lead to impaired immune responses or neurodegeneration.^2^, ^3^, ^4^ Recently, a novel role in the regulation of homeostasis has been ascribed to Plg; namely, Plg binds to the surface of apoptotic cells^5^, ^6^ and thereby induces their clearance by resident macrophages in the process of efferocytosis.^7^ However, how phagocytes recognize Plg exposed on apoptotic cells has remained unknown.

We have previously demonstrated that the mannose 6‐phosphate/insulin‐like growth factor 2 receptor (M6P/IGF2R, CD222) binds and internalizes Plg.^8^, ^9^ M6P/IGF2R is an ubiquitously expressed type I transmembrane glycoprotein that is largely present in the Golgi apparatus and endosomes, and to a minor extent, also on the cell surface.^10^ M6P/IGF2R has an essential role in the selective delivery of both newly synthesized enzymes and extracellular ligands bearing the recognition marker mannose 6‐phosphate (M6P) to lysosomes.^11^ In an M6P‐independent manner, it internalizes insulin‐like growth factor 2 (IGF2), a potent mitogen.^12^ We have pinpointed that M6P/IGF2R via its amino‐terminal region binds^8^ and internalizes Plg.^9^ The crystal structure of this region, resolved by Olson and colleagues, has provided further insights into the shape of this Plg‐binding site.^13^

Here, we show that M6P/IGF2R expression increases during monocyte differentiation to macrophages. Genetic knockdown and knockout of M6P/IGF2R leads to the down‐regulation of Plg‐induced efferocytosis, while reconstitution of M6P/IGF2R expression restores the phagocytosis of apoptotic cells. The level of uptake of Plg‐coated apoptotic cells inversely correlates with the TNF‐α production by phagocytes. Blockade of the Plg‐M6P/IGF2R interaction by an anti‐M6P/IGF2R mAb reduces Plg‐induced efferocytosis. Thus, these results unravel the functional association between M6P/IGF2R and Plg in efferocytosis.

## 2 MATERIALS AND METHODS

### 2.1 Materials

Ammonium persulfate, TEMED, sodium dodecyl sulfate (SDS), acrylamide, and *N*,*N*´‐methylenebisacrylamide were purchased from SERVA (Heidelberg, Germany). Human uPA and Glu‐type plasminogen (Glu‐Plg) were products of Calbiochem (Darmstadt, Germany). BSA was from Carl Roth (Karlsruhe, Germany). Beriglobin was from CSL Behring (King of Prussia, PA, USA). Polybrene, puromycin, and tranexamic acid (TA) were from Sigma–Aldrich (St. Louis, MO). Nonidet P‐40 was obtained from Pierce Biotechnology Inc. (Rockford, IL, USA). Annexin V‐Pacific blue was from BioLegend (San Diego, CA, USA).

### 2.2 Antibodies

The mAbs to M6P/IGF2R (CD222) MEM‐238, biotinylated MEM‐238, and MEM‐240 were generated and produced by us.^14^ The Alexa‐Fluor (AF)‐647‐ and PE‐conjugated MEM‐238, Pacific Blue‐conjugated MEM‐18 mAb (CD14), and isotype‐control AF‐647‐labeled MOPC‐21 and AFP‐01 mAb were from EXBIO Praha (Vestec, Czech Republic). Both mAbs to Plg (4Pg, 7Pg) were from Technoclone (Vienna, Austria). The HRP‐conjugated goat anti‐mouse IgG secondary antibody was from Sigma–Aldrich. Goat anti‐mouse IgG+IgM (H+L)‐FITC conjugate was from An der Grub, Kaumberg, Austria; anti‐mouse IgG conjugates labeled fluorescently with AF‐488 or AF‐647 were from Molecular Probes (Invitrogen Life Technologies, Carlsbad, CA).

### 2.3 Cells

The human monocytic cell line THP‐1 and the human T cell line Jurkat E6.1, both from ATCC were cultured in RPMI‐1640 medium (Invitrogen) supplemented with 100 U/ml penicillin, 100 μg/ml streptomycin, 2 mmol/l l‐glutamine and 10% heat‐inactivated FCS (Sigma–Aldrich). When needed, apoptosis was induced by adding 200 ng/ml staurosporine (SSP). All the cells were grown in a humidified atmosphere at 37°C and 5% CO_2_. All THP‐1 cells, i.e., control‐ and M6P/IGF2R‐silenced via RNAi, control‐ and M6P/IGF2R zinc finger nuclease (ZFN)‐knockout cells, and M6P/IGF2R‐knockout cells with reconstituted M6P/IGF2R expression, were differentiated to the phagocytic phenotype during 48 h culture with 5 ng/ml PMA. Mouse *M6P/IGF2R* ^−/−^ fibroblasts were provided by Dr. E. Wagner (until 2008 ‐ IMP, Vienna, Austria; now CNIO, Madrid, Spain). *M6P/IGF2R* ^−/−^ fibroblasts stably expressing human M6P/IGF2R were prepared by retroviral infection.^8^, ^9^, ^15^, ^16^, ^17^ Mouse M6P/IGF2R‐negative mouse fibroblasts with or without expression of human M6P/IGF2R were used without any stimulation.

Peripheral blood of healthy donors, obtained from the blood bank of the Medical University of Vienna was separated into plasma, PBMCs, and granulocytes with red blood cells using the Lymphoprep gradient centrifugation (Axis Shield, Oslo, Norway). Monocytes were further purified from the PBMC fraction using CD14 MACS microbeads (Miltenyi Biotec, Bergisch Gladbach, Germany). Primary macrophages were differentiated from CD14^+^ monocytes with recombinant human macrophage colony‐stimulating factor (M‐CSF; from Peprotech, Rocky Hill, NJ; 50 ng/ml) as previously described.^18^, ^19^ Following a 7‐day differentiation, macrophages were rested in macrophage serum free medium (Invitrogen), supplemented with 2 mmol/l l‐glutamine and 2% FCS for 2 days prior to efferocytosis assays.

### 2.4 Flow cytometry and cell surface binding assay

Cells were harvested, washed with PBS containing 1% BSA and 0.02% sodium azide, blocked with 2% beriglobin for 15 min on ice and afterward incubated for 30 min on ice with a specific mAb, either fluorescently labeled or unlabeled. For the latter, a second step staining was done with FITC‐conjugated F(ab’)2 anti‐mouse IgG+IgM antibodies (An der Grub, Kaumberg, Austria). Prior to analysis, the cells were washed again and briefly incubated with DAPI. Apoptotic cells were discriminated by staining with DAPI and the Annexin V‐Pacific Blue conjugate (BioLegend). Flow cytometry was performed with an LSR II flow cytometer (Becton Dickinson). Data acquisition was executed with the FACS DIVA software. Data analysis was accomplished with the FlowJo software (Treestar Inc., Ashland, OR).^20^

### 2.5 RNA interference, ZFN, and retroviral transduction technology

The stable knockdown of M6P/IGF2R in THP‐1 by RNAi was generated by the delivery of a short hairpin RNA expression cassette as described in detail elsewhere.^9^, ^21^ Accordingly, we applied 2 constructs targeting M6P/IGF2R mRNA, i.e., at the position 6588 (shM6P/IGF2R‐1) and 4525 (shM6P/IGF2R‐2).^9^, ^21^ As a control construct, the MISSION nontarget shRNA control vector (pLKO.1 puro) from Sigma–Aldrich was applied.

ZFN‐based gene knockout was performed as previously described.^15^ Briefly, Cys2His2‐based zinc fingers were created to specifically target the genomic sequence of *M6P/IGF2R*. The ZFN cassettes were cloned into pMLM290/pMLM292 and pMSM800/pMLM802 (both from Addgene) and these constructs were electroporated to THP‐1 cells using Amaxa Nucleofector (Lonza, Basel, Switzerland). After cell recovery and expansion, M6P/IGF2R‐knockout cells were repeatedly sorted for M6P/IGF2R‐negative cells with an FACSAria (BD) and expanded. These cells are further referred to as THP‐1 KO‐M6PR, while the cells prepared with nontargeting ZFN (empty) vectors, and expanded, are referred to as THP‐1 KO‐CTR.

The recombinant human *M6P/IGF2R* gene was transduced retrovirally into the THP‐1 KO‐M6PR cells as described,^8^ generating THP‐1 REC‐M6PR cells. Briefly, the retroviral expression vector pBMN‐Z was used for transient transfection of the ecotropic packaging cell line Phoenix (both provided by G. Nolan, Stanford University School of Medicine, Stanford, CA, USA). The supernatants containing viral particles were mixed with polybrene (4 μg/ml) to transduce the target cells, i.e., THP‐1 KO‐M6PR cells. The expression of the recombinant M6P/IGF2R was verified by flow cytometry with a specific mAb, PCR, and Western blotting.

### 2.6 RNA isolation and real‐time PCR

Total RNA was extracted from the cells with TRIzol reagent (Invitrogen) supplemented with β‐mercaptoethanol for RNAse inhibition. cDNA was synthesized from 500 ng total RNA using SuperScript III reverse transcriptase (Invitrogen). Quantitative PCR was carried out in duplicates using the TaqMan® Gene Expression Assay System (Invitrogen) in a CFX96 Touch Real Time PCR Detection System (Bio‐Rad, Hercules, CA). To measure *M6P/IGF2R* expression, probe set Hs00974500_m1 was used, together with the probe set Hs03044281_g1 for the endogenous gene *YWHAZ* and analyzed by the 2^–ΔΔCT^ method.^22^ Results are reported relative to the values for one of the monocyte samples, which were set to 1.

### 2.7 Efferocytosis assay

As phagocytic cells, we used primary monocyte‐derived macrophages, THP‐1 cell‐derived macrophages, both control and cells with manipulated expression of M6P/IGF2R as described above, and *M6P/IGF2R* ^−/−^ mouse fibroblasts with or without ectopically expressed human M6P/IGF2R. To generate apoptotic cells, Jurkat T cells were labeled with CFSE (Molecular Probes, Invitrogen, Carlsbad, CA) or alternatively, with eFluor 670 (eBioscience, Thermo Fisher Scientific, Waltham, CA), and apoptosis was induced by SSP (200 ng/ml) for 16 or 9 h. Apoptotic T cells were then pretreated for 30 min with Plg (100 nmol/l), washed with PBS, and cultured with the phagocytic cells at a 5:1 (T cell: phagocyte) ratio. Efferocytosis proceeded for 2–4 h at 37°C in the absence and presence of the indicated molecules (TA 5 mmol/l; mAbs MEM‐238 and MEM‐240 to M6P/IGF2R, mAbs 4Pg and 7Pg to Plg, and control mAb AFP‐01, all at 5 μg/ml). For the mAbs experiments, the cells were co‐treated with 2% beriglobin to block Fc receptors. Afterward, the phagocytes fed with the CFSE‐labeled apoptotic cells were thoroughly washed from the non‐uptaken apoptotic Jurkat T cells and then harvested by trypsinization. By this process, any bound apoptotic cell should be cleaved off from the phagocytes’ surface. Flow cytometry was used to quantify the percentage of cells that phagocytosed apoptotic cells labeled with either CFSE or eFluor 670.

### 2.8 TNF‐α measurement

THP‐1 macrophages (control, THP‐1 KO‐M6PR and THP‐1 REC‐M6PR cells) were subjected to efferocytosis with apoptotic Jurkat T cells at a 2:1 (T cell: phagocyte) ratio in the presence of Plg for 24 h. The cell‐free culture supernatant was then harvested and stored at −80°C. TNF‐α was measured from the supernatants by the Luminex technology as done previously.^23^

### 2.9 Confocal microscopy

Confocal microscopy was performed using the Leica TCS SP8 STED‐3X system equipped with an inverted microscope Leica DMi8, using an HC PL APO CS2 100×/1.40 OIL objective. A diode laser with 405 nm excitation was used for UV excited dyes, all other fluorophores were excited using a white pulsed laser and the emission detection wavelengths were selected using a Leica SP detector. CFSE (green) was excited at 495 nm and emission was collected at 505–560 nm. AF647 and eFluor 670 were excited at 647 nm and emission was collected at 660–710 nm. PE was excited at 560 nm and emission was collected at 570–620 nm. DAPI emission was collected from 415 to 470 nm. Z‐stacks were taken in slices of 300 nm. The primary human macrophages were harvested after an above‐described 7‐day differentiation and cultured for 2 days directly on microscope slides in macrophage serum free medium, supplemented with 2 mmol/l l‐glutamine and 2% FCS. In one setting, after the efferocytosis assay with the CFSE‐ or eFluor 670‐labeled apoptotic Jurkat T cells the macrophages or fibroblasts were fixed with 4% formaldehyde, permeabilized with 0.1% saponin, blocked with 2% beriglobin, and stained with AF647‐conjugated M6P/IGF2R mAb MEM‐238 in PBS containing 2% BSA. Nuclei were stained with DAPI. The slides were washed with PBS and mounted with mounting medium for fluorescence analysis (Vectashield; Vector Laboratories, Burlingame, CA). In the other setting, the macrophages on the slides were first preincubated for 30 min with 2% beriglobin, followed by the mAb MEM‐238‐AF647 for 30 min. Then, macrophages were washed with the medium and subjected to the efferocytosis assay with the CFSE‐positive apoptotic cells for 2 h, washed, and analyzed. The images were analyzed by using the Leica software acquired with the microscope and the open‐source software ImageJ.

### 2.10 Statistical analysis

All experiments were performed at least 3 times in at least triplicates. The data were expressed as mean values with SD. Statistical significance was evaluated by using a Student's *t*‐test; a value of ^*^ *p* < 0.05, ^**^ *p* < 0.005, or ^***^ *p* < 0.0005 was considered to be significant or highly significant, respectively. Analysis and graphing were performed using GraphPad Prism 5 (GraphPad Software, San Diego, CA).

## 3 RESULTS

**Figure 1:**
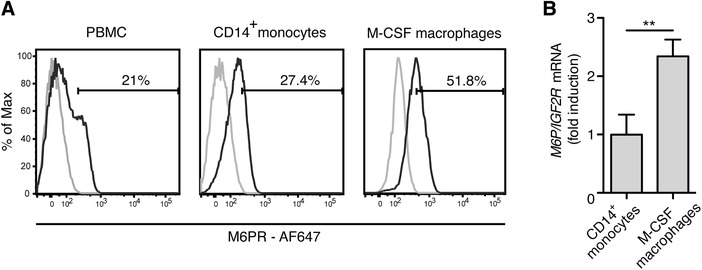
**M6P/IGF2R expression increases during monocyte differentiation to macrophages**. (**A**) Cell‐surface expression of M6P/IGF2R on isolated PBMC and MACS‐enriched monocytes from healthy donors was evaluated with mAb MEM‐238‐AF647 and flow cytometry. In parallel, MOPC‐21‐AF647 was used as an isotype control mAb, displayed by the cut‐off gates. The same analysis was performed with macrophages differentiated from MACS‐sorted monocytes during a 7‐day culture with recombinant human M‐CSF (50 ng/ml) followed by 2 days resting in macrophage serum free medium. (**B**) Primary human monocytes and monocyte‐derived macrophages from (A) were lysed and RNA was extracted. cDNA was synthesized from total RNA and gene expression was measured by real‐time PCR as described in the Material and Methods section with TaqMan primer sets for human *M6P/IGF2R* and *YWHAZ* as endogenous control. The *M6P/IGF2R* mean expression values relative to that of monocytes ± SD from 3 donors is shown

By staining PBMCs, we found very low surface expression of M6P/IGF2R. Only about 20% of PBMC displayed markedly higher M6P/IGF2R surface expression (Fig. 1A). We identified this M6P/IGF2R^+^ subpopulation as CD14^+^ monocytes (Fig. 1A). The M6P/IGF2R surface expression increased when we differentiated the primary monocytes toward macrophages using M‐CSF (Fig. 1A) and this increase was also recapitulated at the level of mRNA (Fig. 1B). Based on this, we concluded that the overexpression of M6P/IGF2R on differentiated macrophages was regulated at the level of transcription.

**Figure 2:**
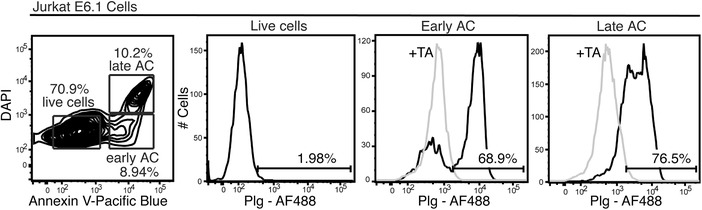
**Plg marks apoptotic cells**. Jurkat T cells were stained on ice with Plg‐AF647, Annexin V‐Pacific blue and DAPI, and analyzed by flow cytometry to discriminate early (Annexin V^+^) and late (Annexin V^+^ / DAPI^+^) apoptotic cells (AC) from viable (Annexin V^−^ / DAPI^−^) cells. Optionally, we co‐incubated the cells with Plg‐AF647 and TA (5 mmol/l)

We showed earlier that M6P/IGF2R binds and internalizes Plg and thereby regulates the proteolytic activity of this powerful enzyme.^8^, ^9^ Because Plg efficiently coats apoptotic cells,^5^, ^6^, ^7^ we asked whether another function of M6P/IGF2R might be the Plg‐mediated efferocytosis of apoptotic cells by macrophages. In our first experiment, we analyzed if Plg bound specifically to apoptotic cells also in our hands. By means of flow cytometric analysis allowing a discrimination of apoptotic from viable cells via the combined staining with Annexin V and DAPI, we observed a strong and specific binding of Alexa Fluor (AF)‐488 conjugated Plg to apoptotic but not to viable Jurkat T cells (Fig. 2). We observed similar results with Annexin V and propidium iodide co‐staining (data not shown). The binding of Plg to apoptotic cells was completely blocked in the presence of tranexamic acid (TA), a lysine analogue that blocks Plg binding to Plg receptors, suggesting that lysine‐binding sites within kringle domains were implicated in the binding of Plg to apoptotic cells (Fig. 2).

**Figure 3:**
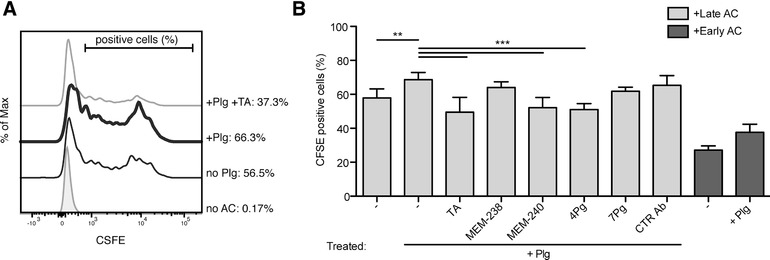
**Flow cytometry analysis of Plg‐mediated efferocytosis by human macrophages**. (**A**) A representative flow cytometry histogram of the efferocytosis analysis. Jurkat T cells were fluorescently labeled with CFSE and their apoptosis was induced by SSP treatment (200 ng/ml) for 16 h. Then, the apoptotic cells (AC) were pretreated for 30 min with or without Plg (100 nmol/l) and TA (5 mmol/l), washed, and added to monocyte‐derived macrophages (generated as in Fig. 1). Incubation was performed for 2 h at 37°C at the macrophage:apoptotic cell ratio of 1:5; without Plg (thin black line), with Plg (bold black line), with Plg and TA (thin grey line). (**B**) Flow cytometry was used to quantify percentages of macrophages that phagocytosed CFSE‐labeled apoptotic cells. The levels of efferocytosis are displayed as percentages of CFSE‐positive macrophages. Incubation was performed for 2 h at 37°C at the macrophage:apoptotic cell ratio of 1:5 in the presence of the following additives: Plg, 100 nmol/l, TA, 5 mmol/l, mAbs MEM‐238 and MEM‐240 to M6P/IGF2R, mAbs 4Pg and 7Pg to Plg, and control mAb AFP‐01, all 5 μg/ml. In some experiments, apoptosis of the Jurkat cells was induced by SSP treatment (200 ng/ml) for 9 h (in dark grey). Mean ± SD of at least 5 independent experiments is shown

Based on these observations, we examined the role of M6P/IGF2R in the uptake of Plg‐coated apoptotic cells. We co‐cultured M‐CSF‐differentiated human macrophages with CFSE‐labeled apoptotic Jurkat T cells and evaluated efferocytosis by flow cytometry (Fig. 3). Since the late apoptotic cells displayed more binding of Plg than the early apoptotic cells (Fig. 2), we induced apoptosis of Jurkat cells by treatment with SSP for as long as 16 h. Approximately 55% of human primary macrophages engulfed apoptotic cells; strikingly, efferocytosis was significantly increased by pre‐incubation of apoptotic cells with Plg (100 nmol/l), where, on average, 70% of the macrophages engulfed CFSE‐labeled Jurkat T cells. TA (5 mmol/l) dampened Plg‐induced efferocytosis (Fig. 3A and B) similarly to the anti‐M6P/IGF2R mAb MEM‐240, but not mAb MEM‐238 recognizing a different epitope on M6P/IGF2R (Fig. 3B). We found the same pattern with the anti‐Plg mAbs: 4Pg inhibited efferocytosis whereas 7Pg, recognizing a different epitope on Plg, did not (Fig. 3B). The mAb MEM‐240 recognizes an epitope within the extracellular repeat domains 6 to 9 of M6P/IGF2R^14^ and mAb 4Pg an epitope within the catalytic part of Plg.^24^ We were able previously to coprecipitate the Plg–M6P/IGF2R complex from human serum with these two mAbs,^16^ suggesting that they do not interfere with the Plg–M6P/IGF2R binding but are able, maybe due to steric hindrance, to inhibit the efferocytosis process.

We performed the efferocytosis experiments also with early apoptotic cells that we generated by treatment of Jurkat T cells with SSP for 9 h. Their uptake was 50% compared to late apoptotic cells (Fig. 3B, right). However, the positive effect of Plg pretreatment on efferocytosis was again substantial (increase from 25 to 40% macrophages that engulfed Plg‐labeled apoptotic cell bodies). Our data are in line with a previous report showing that Plg binding to apoptotic cells exhibits slightly delayed kinetics compared to the phosphatidylserine exposure on the apoptotic cell surface.^6^

**Figure 4:**
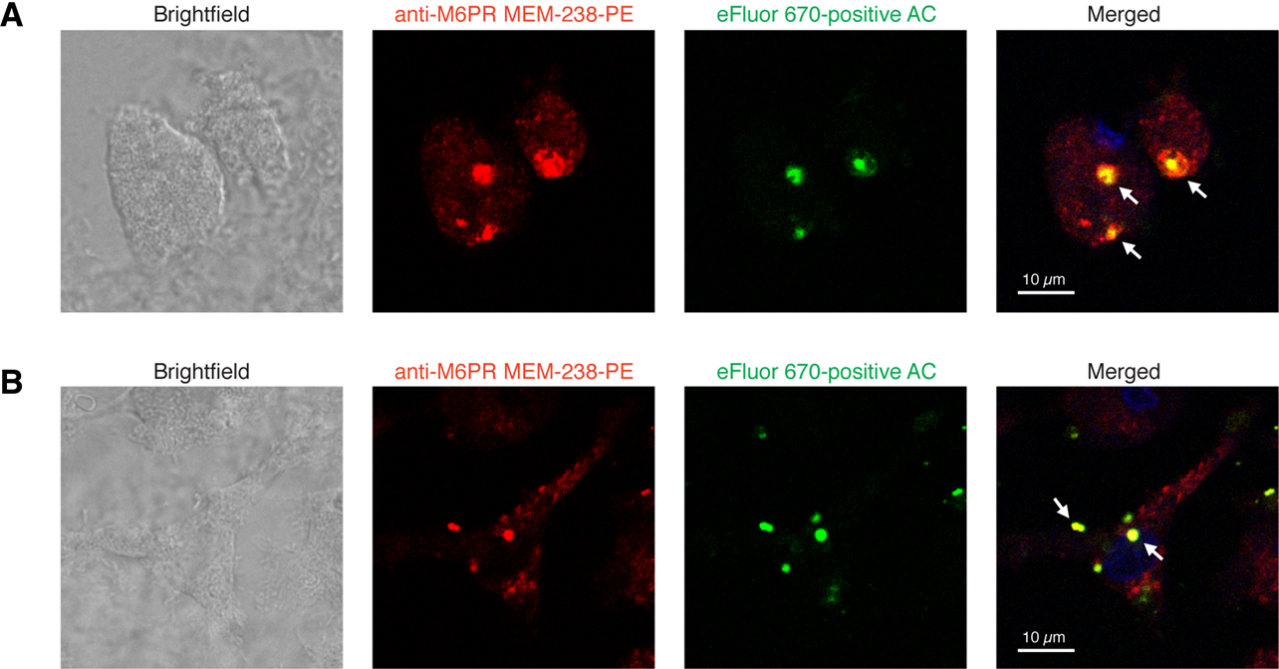
**Confocal microscopy analysis of Plg‐mediated efferocytosis by human macrophages**. (**A**) After incubation with eFluor 670‐labeled apoptotic Jurkat T cells (green) for 2 h the macrophages were fixed, permeabilized and stained with PE‐conjugated M6P/IGF2R mAb MEM‐238 (red). Nuclei were stained with DAPI (blue). The slides were washed and analyzed by confocal microscopy. (**B**) Macrophages grown on microscope slides were preloaded for 30 min with mAb MEM‐238‐PE, washed and subjected to the efferocytosis assay with eFluor 670‐labeled apoptotic Jurkat cells for 2 h. Then the slides were washed and analyzed by confocal microscopy; the scales represent 10 μm; arrows point to the region of colocalization of M6P/IGF2R and apoptotic cells (AC)

We analyzed the engulfment of apoptotic cells by macrophages also by confocal microscopy. We fixed and stained macrophages with fluorescently labeled nonblocking anti‐M6P/IGF2R mAb MEM‐238 and analyzed the staining in relation to CFSE‐positive apoptotic cell bodies. We detected zones of colocalization of engulfed apoptotic bodies and M6P/IGF2R (Fig. 4A). The 3D cell analysis also showed intracellular colocalization of M6P/IGF2R with CFSE‐positive engulfed apoptotic cells (Supplementary Fig. 1). Next, we analyzed living macrophages preloaded with labeled MEM‐238 mAb. In this setting, adherent differentiated macrophages were preincubated for 30 min with anti‐M6P/IGF2R mAb MEM‐238‐PE, then subjected to the efferocytosis assay with the CFSE‐positive apoptotic cells for 2 h, washed on a plate, and analyzed immediately. Within macrophages, fragments of CFSE‐positive apoptotic cells could be recognized, which colocalized with the internalized MEM‐238 (Fig. 4B). All these findings indicate a common endosomal/lysosomal pathway for engulfed apoptotic cells and M6P/IGF2R.

**Figure 5:**
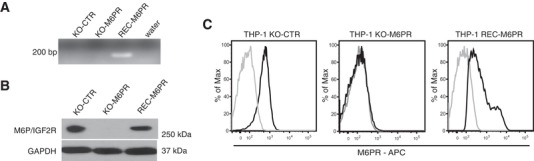
**Generation of M6P/IGF2R‐knock‐out and ‐reconstituted THP‐1 cells**. Knockout of M6P/IGF2R in THP‐1 cells was performed via targeting the *M6P/IGF2R* gene in the Exon 22 on chromosome 6 by means of the Zinc finger nuclease (ZFN) technology. The expression of M6P/IGF2R was reconstituted in the knockout cells through retroviral transduction of the DNA fragment encoding human M6P/IGF2R. The expression of M6P/IGF2R was verified at both the gene and the protein level. (**A**) Genomic DNA purified from THP‐1 control cells (KO‐CTR), M6P/IGF2R‐knock‐out cells (KO‐M6PR), and KO‐M6PR cells reconstituted with M6P/IGF2R (REC‐M6PR) was used as a template for the PCR amplification with exon‐crossing primer sets for human *M6P/IGF2R* recognizing the recombinant *M6P/IGF2R* gene. (**B**) The total M6P/IGF2R contents in control cells (KO‐CTR), M6P/IGF2R‐knock‐out THP‐1 cells (KO‐M6PR) and KO‐M6PR cells reconstituted with human M6P/IGF2R (REC‐M6PR) were analyzed by Western blotting using anti‐M6P/IGF2R mAb MEM‐238. GAPDH served as a loading control. (**C**) M6P/IGF2R surface expression (black) of control, M6P/IGF2R‐knockout and ‐reconstituted THP‐1 cells was analyzed via flow cytometry with mAb MEM‐238‐AF647. The isotype control mAb staining is shown in light grey

To test whether M6P/IGF2R was specifically responsible for the clearance of Plg‐coated apoptotic cells, we employed the human monocytic THP‐1 cell line. THP‐1 cells display a relatively high surface expression of M6P/IGF2R^9^ (Fig. 5) and are capable of phagocytosis when differentiated.^25^ We modified THP‐1 cells by means of genetic knockdown, knockout, and rescue approaches: First, we used control and M6P/IGF2R‐silenced THP‐1 cells that had been previously generated by RNA interference (RNAi)^9^; second, via sequence‐specific ZFNs, we generated a genetic M6P/IGF2R knockout in THP‐1 cells that was, third, rescued by retroviral transduction of the recombinant human M6P/IGF2R construct (Fig. 5A–C), as described previously for Jurkat T cells.^15^

**Figure 6:**
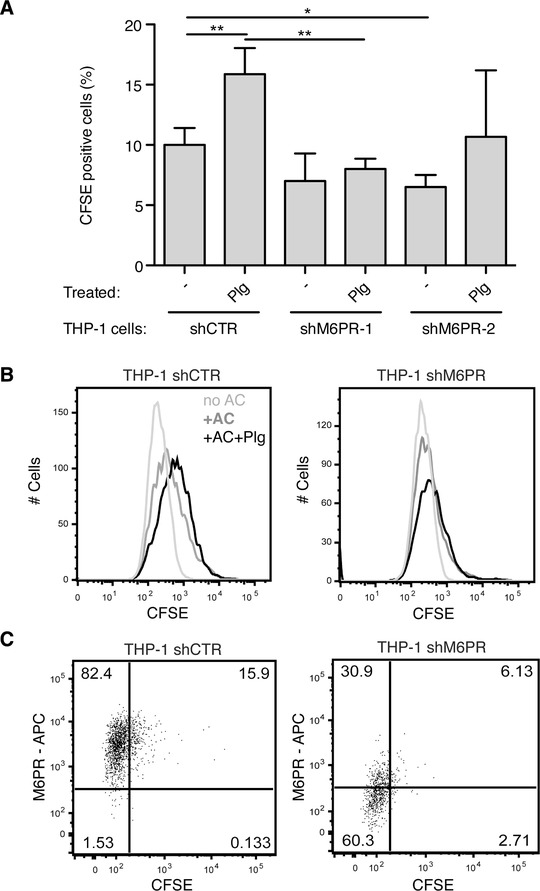
**Genetic knockdown of M6P/IGF2R affects Plg‐triggered efferocytosis**. (**A**) Jurkat T cells were fluorescently labeled with CFSE and apoptosis was induced by SSP (200 ng/ml) for 16 h. Then, the apoptotic cells were pretreated for 30 min with Plg (100 nmol/l), washed, and added to the THP‐1 cells differentiated to a phagocytic phenotype with PMA (5 ng/ml, 48 h). The THP‐1 cells were derived from control and two M6P/IGF2R‐knockdown populations: THP‐1 shCTR, THP‐1 shM6PR‐1, and THP‐1 shM6PR‐2. Efferocytosis was performed for 4 h at 37°C at the phagocyte:apoptotic cell ratio of 1:5 optionally in the presence of Plg (100 nmol/l). Flow cytometry was used to quantify percentages of macrophages that phagocytosed CFSE‐labeled apoptotic cells. The levels of efferocytosis are displayed as percentages of CFSE‐positive phagocytes. Mean ± SD of at least 4 independent experiments is shown. (**B**) Representative flow cytometry histograms of the efferocytosis analysis. Efferocytosis was performed as in (A) with control‐ and M6P/IGF2R‐knockdown THP‐1 cells (shM6PR‐1) differentiated to a phagocytic phenotype with PMA (5 ng/ml, 48 h) in the presence of anti‐M6P/IGF2R mAb MEM‐238‐AF647 (5 μg/ml) and Plg (100 nmol/l). After the assay, the plate was washed, the adherent phagocytes were harvested by trypsin and analyzed by flow cytometry. (**C**) Representative flow cytometry dot plots of the efferocytosis analysis. Efferocytosis was performed as in (B) with Plg

We differentiated the various THP‐1 cells to phagocytes by using PMA and subjected the cells to the efferocytosis assay with apoptotic CFSE‐labeled Jurkat T cells. These cells displayed, in contrast to the primary macrophages, a 5 times lower capacity to engulf apoptotic cells—50% vs. 10% (Fig. 6A). M6P/IGF2R silencing at positions 6588 (shM6PR‐1) and 4525 (shM6PR‐2) led to a decrease in efferocytosis, as only ∼6% of M6P/IGF2R‐silenced THP‐1 cells contained CFSE‐labeled apoptotic bodies (Fig. 6A–C). Moreover, the efferocytosis capacity of the M6P/IGF2R‐silenced cells did not increase when the apoptotic cells were pretreated with Plg. In contrast, the control cells displayed a 50% increase of clearance of apoptotic cells upon Plg pretreatment (Fig. 6A and B). When we co‐incubated the THP‐1 phagocytes with fluorescently labeled nonblocking anti‐M6P/IGF2R mAb MEM‐238‐AF647, cells displaying a higher engulfment of CFSE‐labeled apoptotic cells exhibited also a higher uptake of MEM‐238‐AF647, indicating that M6P/IGF2R internalization correlated positively with the phagocytic potential (Fig. 6C).

**Figure 7:**
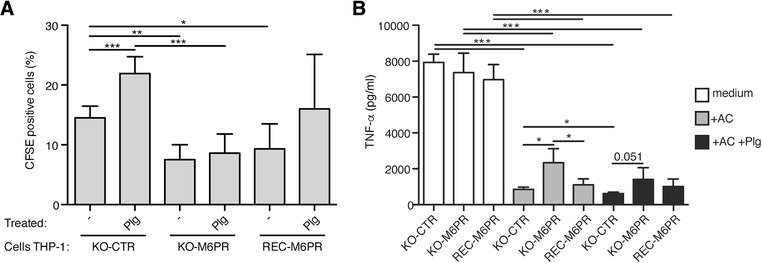
**Genetic knockout and reconstitution of M6P/IGF2R affects Plg‐triggered efferocytosis and TNF‐α secretion in THP‐1 derived phagocytes**. (**A**) Jurkat T cells were fluorescently labeled with CFSE and apoptosis was induced by SSP (200 ng/ml) for 16 h. The apoptotic cells (AC) were pretreated or not for 30 min with Plg (100 nmol/l), washed, and added to THP‐1 cells differentiated to a phagocytic phenotype with PMA (5 ng/ml, 48 h). THP‐1 cells were either control knockout, M6P/IGF2R‐knockout, or the latter reconstituted with human M6P/IGF2R: THP‐1 KO‐CTR, THP‐1 KO‐M6PR, and THP‐1 REC‐M6PR. Efferocytosis was performed for 4 h at 37°C at the phagocyte:apoptotic cell ratio of 1:5 optionally in the presence of Plg (100 nmol/l). Flow cytometry was used to quantify percentages of macrophages that phagocytosed CFSE‐labeled apoptotic cells. The levels of efferocytosis are displayed as percentages of CFSE‐positive phagocytes. Mean ± SD of at least 4 independent experiments is shown. (**B**) THP1 phagocytes from (A) were either left untreated (shown in white) or incubated with apoptotic Jurkat cells (in grey) or Plg‐pretreated apoptotic Jurkat cells (in black) at a ratio 1:2. After 24 h incubation, cell‐free medium was harvested and TNF‐α was measured by the Luminex technology. A representative of 3 independent experiments is shown, where mean concentrations ± SD from cocultures performed in quadruplicates were measured

Similar to the M6P/IGF2R knockdown cells, also M6P/IGF2R‐knockout THP‐1 cells showed a 50% decreased efferocytosis. Further, these cells did not engulf more apoptotic cells when we pretreated the latter with Plg, in contrast to THP‐1 KO‐CTR cells where Plg induced a ∼50% increase in efferocytosis (Fig. 7A). However, when we re‐expressed recombinant human M6P/IGF2R in the M6P/IGF2R‐knockout cells, the reconstituted cells showed again the increased uptake of Plg‐pretreated apoptotic cells as the control THP‐1 phagocytes (Fig. 7A).

Engulfment of apoptotic cells is known to reprogram macrophages from a proinflammatory to a pro‐resolution state, characterized by suppression of pro‐inflammatory cytokine production (e.g., TNF‐α, IL‐12, and IL‐1β) and potentiation of secretion of anti‐inflammatory cytokines IL‐10, TGF‐β, and other pro‐resolving mediators.^26^, ^27^, ^28^ To ascertain the role of M6P/IGF2R in the aforementioned macrophage reprogramming, we measured TNF‐α levels from supernatants of the THP‐1 phagocytes that had been fed (or not) with apoptotic Jurkat cells (Fig. 7B). Under control conditions, TNF‐α was potently produced by PMA‐differentiated THP‐1 phagocytes, in agreement with published data.^29^ However, the cocultivation of THP‐1 KO‐CTR cells with apoptotic cells led to a robust, nearly 90% decrease in TNF‐α production. Upon M6P/IGF2R‐knockout, the suppression of TNF‐α production was less pronounced, resulting in approximately 68% decrease, while re‐expression of M6P/IGF2R in the M6P/IGF2R knockout cells was associated with more powerful suppression of TNF‐α (84% decrease; Fig. 7B, middle). The Plg‐pretreatment of apoptotic cells led to even more pronounced suppression of TNF‐α production with a similar pattern observed with non‐pretreated apoptotic cells (Fig. 7B, right).

**Figure 8:**
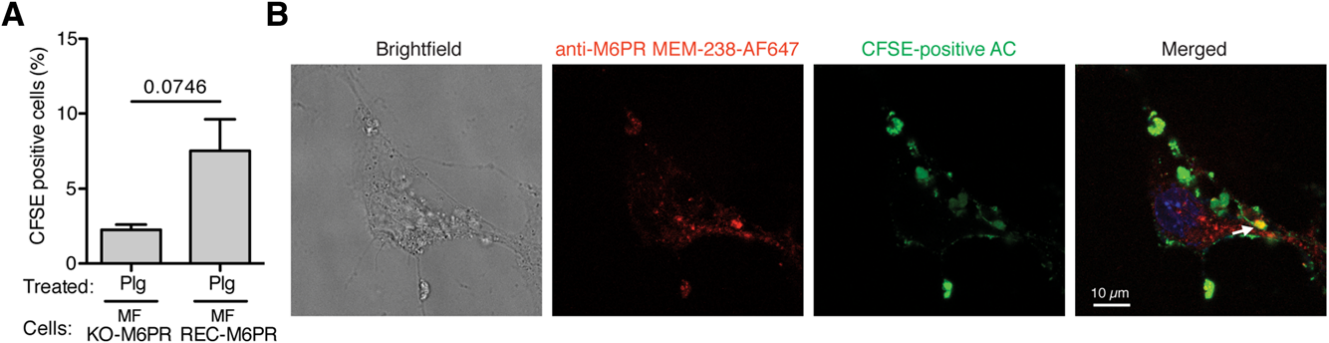
**Reconstitution of M6P/IGF2R affects Plg‐triggered efferocytosis in mouse fibroblasts**. (**A**) Apoptotic Jurkat T cells were pretreated for 30 min with Plg (100 nmol/l), washed, and added to M6P/IGF2R‐negative mouse fibroblasts (MF KO‐M6PR) or M6P/IGF2R‐negative mouse fibroblasts transduced with human M6P/IGF2R (MF REC‐M6PR). Efferocytosis was performed as in Fig. 7. Flow cytometry was used to quantify percentages of macrophages that phagocytosed CFSE‐labeled apoptotic cells. The levels of efferocytosis are displayed as percentage of CFSE‐positive phagocytes. Mean ± SD of at least 5 independent experiments is shown. (**B**) After the efferocytosis assay with CFSE‐labeled apoptotic Jurkat T cells (green), mouse fibroblasts expressing human M6P/IGF2R were fixed, permeabilized and stained with AF647‐conjugated M6P/IGF2R mAb MEM‐238 (red). The slides were washed and analyzed using confocal microscopy. An arrow points to the region of colocalization of M6P/IGF2R and apoptotic cells

Finally, we verified our data also with fibroblasts, because Hall and colleagues showed that fibroblasts, although non‐professional phagocytes, were also able to engulf apoptotic cells.^28^, ^30^ Furthermore, we reported previously that re‐expression of human M6P‐IGF2R in mouse fibroblasts derived from M6P‐IGF2R knockout mice resulted in an increased Plg internalization.^9^ Indeed, these fibroblasts expressing human M6P/IGF2R executed efferocytosis. Although the level of efferocytosis of Plg‐pretreated apoptotic cells in these cells was low (6%), this experiment is a further proof that M6P‐IGF2R is responsible for Plg‐mediated efferocytosis, because the human M6P‐IGF2R‐negative mouse fibroblasts scored negligible (Fig. 8A). We analyzed the engulfment of apoptotic cells by fibroblasts also by confocal microscopy. We fixed and stained the fibroblasts with the directly labeled anti‐M6P/IGF2 mAb MEM‐238 and analyzed the uptake of CFSE‐positive apoptotic cells. Also in this case, the results showed the intracellular colocalization of M6P/IGF2R with the CFSE‐positive fragments of apoptotic cells (Fig. 8B).

## 4 DISCUSSION

Apoptosis, a genetically programmed process of cell death, is an essential physiological process involved in tissue remodeling, hematopoiesis, and inflammation. When dysregulated, it crucially contributes to the development of many pathologies including tumor progression,^31^ neurodegenerative disorders,^32^ or serious inflammatory conditions.^33^ Apoptosis involves many intracellular molecular pathways and culminates in the removal of apoptotic bodies by tissue resident macrophages or other surrounding cells in a process called efferocytosis.^33^ Rapid efferocytosis guarantees that no inflammation is triggered by apoptotic cells and no damage of the tissue occurs, in contrast to necrotic cell death.^31^ Many receptors have been characterized on phagocytes to be involved in efferocytosis. Most of them recognize phosphatidylserine exposed on apoptotic cells either directly, such as the TIM proteins TIM‐1, TIM‐3, and TIM‐4,^34^, ^35^ BAI1,^36^ the CD300 proteins,^37^ stabilin‐1 and stabilin‐2,^38^, ^39^ or indirectly by recognizing phosphatidylserine‐binding bridging serum proteins: α_V_β_3/5_ integrins via binding serum proteins MFG‐E8, CCN1, and vitronectin^40^, ^41^, ^42^ or the TAM tyrosine kinases Tyro3, Axl, and Mertk via binding serum proteins Gas6 and protein S.^43^ In addition, other receptors, such as uPAR,^44^ CD36,^45^ CD91,^46^ or CD14^47^ have been shown to mediate efferocytosis. Another bridging serum protein appears to be Plg, because it binds to apoptotic cells^5^, ^6^ and promotes the clearance of apoptotic cells^6^, ^7^ by functioning as an “eat‐me” signal in vivo.^7^ However, it is not known which one of the Plg receptors on the surface of phagocytes is responsible for the recognition and uptake of Plg‐coated apoptotic cells. Here, we show that it is M6P/IGF2R, a known Plg receptor,^8^, ^9^ which mediates the Plg‐induced efferocytosis.

M6P/IGF2R is a central protein transporter involved in lysosome biogenesis.^10^ Accordingly, it is mostly expressed intracellularly in the Golgi apparatus and endosomal compartments. However, upon activation and/or differentiation, it can be rapidly distributed to the cell surface.^15^, ^48^ In agreement with these findings, we show that M6P/IGF2R surface expression increases upon differentiation of primary monocytes toward macrophages. Cell surface M6P/IGF2R mediates binding and uptake of various extracellular ligands resulting in their degradation in lysosomes^9^, ^49^, ^50^; in addition, as shown by us here it enhances efferocytosis by binding Plg.

Interestingly, enhanced efferocytosis is associated with Plg activation and plasmin activity^6^, ^7^ suggesting that extracellular proteolysis might yield apoptotic “eat‐me” signals recognized by phagocytes.^51^ Although we show here that M6P/IGF2R mediates recognition of Plg‐coated apoptotic cells by macrophages, the identification of Plg receptor(s) on the apoptotic cells remains to be determined. It is most likely a protein, because TA, a lysine analogue that blocks lysine‐dependent binding of Plg, inhibited the Plg binding to apoptotic cells. A plethora of diverse Plg receptors has been characterized that mostly potentiate plasmin generation on the cell surface. Among them is annexin A2 that has been shown to mediate clearance of apoptotic cells by functioning as a ligand for C1q.^52^ Other Plg receptors include cytokeratin 8, dipeptidyl peptidase IV, S100A10, Plg‐R(KT), prion, and others (see Refs. 53, 54, 55, 56, 57, 58). In contrast to them, M6P/IGF2R down‐regulates plasmin generation on the cell surface.^8^, ^9^

Studies in the mouse have demonstrated that the Plg/plasmin system influences macrophages by transcriptional modulation of several genes to increase their efferocytosis activity^7^ and by promoting their reprogramming from the proinflammatory (M1) to the resolution (M2) type.^57^ However, these studies have not identified the Plg receptor on the phagocytes. Here, we show by means of loss‐of‐ and gain‐of‐function genetic approaches that M6P/IGF2R facilitates not only the Plg‐induced efferocytosis but also contributes to the efferocytosis‐mediated macrophage switch by restraining production of the proinfammatory cytokine TNF‐α. The genetic knock‐out of M6P/IGF2R in THP‐1‐derived macrophages abrogates the Plg‐induced efferocytosis and increases the TNF‐α production indicating that M6P/IGF2R contributes significantly to the Plg‐mediated clearance of apoptotic cells and macrophage reprogramming. We have previously shown that M6P/IGF2R internalizes Plg leading to its degradation, and thus serves as a negative regulator of Plg activation.^9^ Thus, the simultaneous apoptotic clearance and the control of the Plg/plasmin system by M6P/IGF2R might ensure that apoptotic cells do not progress to secondary necrosis associated with generation of proteolytically processed neoantigens, which underlies the pathogenesis of chronic inflammatory autoimmune diseases.^27^, ^59^

When we incubated the apoptotic cells with Plg, the phagocytic capacity of the macrophages was enhanced, whereas TA reduced efferocytosis below the basal level. These results indicate that Plg‐mediated efferocytosis occurs also in the basic human Plg‐free setup of the assay. This might be either Plg independent, or due to the bovine Plg present in serum that could have pre‐coated apoptotic bodies, since the bovine Plg molecule can also bind to human M6P/IGF2R.^60^ Further, the genetic knockout of M6P/IGF2R in phagocytes did not eliminate their efferocytic capacity completely suggesting that other receptor(s) were implicated in recognizing exposed phosphatidylserine and/or other “eat‐me” signals on apoptotic cells, or alternatively, other Plg receptors expressed on macrophages might be involved.

In the majority of cell types, the protein transport routes of ubiquitously expressed M6P/IGF2R are mostly restricted to the Trans‐Golgi‐network and endosomal compartments. As few as 5–10% of the M6P/IGF2R molecules are typically displayed on the cell surface,^61^ where they bind and internalize various extracellular ligands, such as IGF2,^62^, ^63^, ^64^ heparanase,^65^, ^66^ leukemia inhibitory factor,^67^ proliferin,^68^, ^69^ TGF‐β,^70^ or Plg.^8^, ^71^ In contrast to Plg, we did not observe that other ligands of M6P/IGF2R, such as IGF2 or M6P, directly affected efferocytosis (data not shown). However, it is possible that through the modulation of surface expression of M6P/IGF2R by specific ligands also Plg‐dependent efferocytosis is modulated.

Surface expression of M6P/IGF2R can change fast upon ligand binding.^72^ On T lymphocytes the surface expression of M6P/IGF2R is very low, although it can be upregulated upon activation.^15^, ^61^ Similarly, low surface expression of M6P/IGF2R was detected on B lymphocytes.^73^ According to our results, among PBMC, monocytes display the highest surface expression of M6P/IGF2R, which can be further increased upon differentiation toward macrophages. Similarly, the monocytic cell line THP‐1 displays relatively high surface M6P/IGF2R. High surface expression on phagocytes and rapid trafficking of M6P/IGF2R within endocytic pathways put M6P/IGF2R as a crucial phagocytic receptor implicated in efferocytosis, and thus as a potential target in disorders accompanied with the unbalanced clearance of apoptotic cells.

## Supporting information


**Figure S1**: Confocal microscopy analysis of Plg‐mediated efferocytosis by human macrophages. After incubation with CFSE‐labeled apoptotic Jurkat T cells (*green*) for 2 h, monocyte‐derived macrophages were fixed, permeabilized and stained with AF647‐conjugated anti‐M6P/IGF2R mAb MEM‐238 (*red*). Nuclei were stained with DAPI (*blue*). The slides were washed and analyzed by confocal microscopy. A 3D confocal image reconstructed from 15 z‐stacks in the total range of 4.18 μm is shown. Arrows point to the region of colocalization of M6P/IGF2R and apoptotic bodies; the scale represents 20 μm.

## References

[1] Dano K , Behrendt N , Hoyer‐Hansen G , et al. Plasminogen activation and cancer. Thromb Haemost. 2005;93:676–681. 15841311 10.1160/TH05-01-0054

[2] Dotti CG , Galvan C , Ledesma MD . Plasmin deficiency in Alzheimer's disease brains: causal or casual?. Neurodegener Dis. 2004;1:205–212. 16908991 10.1159/000080987

[3] Gyetko MR , Aizenberg D , Mayo‐Bond L . Urokinase‐deficient and urokinase receptor‐deficient mice have impaired neutrophil antimicrobial activation in vitro. J Leukoc Biol. 2004;76:648–656. 15240745 10.1189/jlb.0104023

[4] Gyetko MR , Sud S , Chensue SW . Urokinase‐deficient mice fail to generate a type 2 immune response following schistosomal antigen challenge. Infect Immun. 2004;72:461–467. 14688127 10.1128/IAI.72.1.461-467.2004 PMC343962

[5] O'Mullane MJ , Baker MS . Loss of cell viability dramatically elevates cell surface plasminogen binding and activation. Exp Cell Res. 1998;242:153–164. 9665813 10.1006/excr.1998.4067

[6] Rosenwald M , Koppe U , Keppeler H , et al. Serum‐derived plasminogen is activated by apoptotic cells and promotes their phagocytic clearance. J Immunol. 2012;189:5722–5728. 23150713 10.4049/jimmunol.1200922

[7] Das R , Ganapathy S , Settle M , Plow EF . Plasminogen promotes macrophage phagocytosis in mice. Blood. 2014;124:679–688. 24876560 10.1182/blood-2014-01-549659 PMC4118484

[8] Leksa V , Godar S , Cebecauer M , et al. The N terminus of mannose 6‐phosphate/insulin‐like growth factor 2 receptor in regulation of fibrinolysis and cell migration. J Biol Chem. 2002;277:40575–40582. 12189157 10.1074/jbc.M207979200

[9] Leksa V , Pfisterer K , Ondrovicova G , et al. Dissecting mannose 6‐phosphate‐insulin‐like growth factor 2 receptor complexes that control activation and uptake of plasminogen in cells. J Biol Chem. 2012;287:22450–22462. 22613725 10.1074/jbc.M112.339663 PMC3391135

[10] Ghosh P , Dahms NM , Kornfeld S . Mannose 6‐phosphate receptors: new twists in the tale. Nat Rev Mol Cell Biol. 2003;4:202–212. 12612639 10.1038/nrm1050

[11] Olson LJ , Castonguay AC , Lasanajak Y , et al. Identification of a fourth mannose 6‐phosphate binding site in the cation‐independent mannose 6‐phosphate receptor. Glycobiology. 2015. 10.1093/glycob/cwv001 PMC4410830 25573276

[12] Hartman MA , Kreiling JL , Byrd JC , MacDonald RG . High‐affinity ligand binding by wild‐type/mutant heteromeric complexes of the mannose 6‐phosphate/insulin‐like growth factor II receptor. FEBS J. 2009;276:1915–1929. 19236480 10.1111/j.1742-4658.2009.06917.x PMC2748650

[13] Olson LJ , Yammani RD , Dahms NM , Kim JJ . Structure of uPAR, plasminogen, and sugar‐binding sites of the 300 kDa mannose 6‐phosphate receptor. Embo J. 2004;23:2019–2028. 15085180 10.1038/sj.emboj.7600215 PMC424385

[14] Godár S , Leksa V , Cebecauer M , Hilgert I , Horejsi V , Stockinger H . CD222 (Mannose‐6 phosphate/insulin‐like growth factor II‐receptor) summary and workshop report In: Mason D, Andre P, Bensussan A, Buckley C, Civin C, Clark E, de Haas M, Goyert S, Hadam M, Hart D, Horejsi V, Jones Y, Meuer S, Morrissey J, Schwarz‐Albiez R, Shaw S, Simmons D, Turni L, Uguccioni M, van der Schoot E, Vivier E, and Zola H, eds. Leukocyte Typing VII. Oxford, UK: Oxford University Press; 2002:482–485.

[15] Pfisterer K , Forster F , Paster W , et al. The late endosomal transporter CD222 directs the spatial distribution and activity of Lck. J Immunol. 2014;193:2718–2732. 25127865 10.4049/jimmunol.1303349

[16] Leksa V , Loewe R , Binder B , et al. Soluble M6P/IGF2R released by TACE controls angiogenesis via blocking plasminogen activation. Circ Res. 2011;108:676–685. 21273553 10.1161/CIRCRESAHA.110.234732

[17] Leksa V , Godar S , Schiller HB , et al. TGF‐{beta}‐induced apoptosis in endothelial cells mediated by M6P/IGFII‐R and mini‐plasminogen. J Cell Sci. 2005;118:4577–4586. 16179614 10.1242/jcs.02587

[18] Machacek C , Supper V , Leksa V , et al. Folate receptor beta regulates integrin CD11b/CD18 adhesion of a macrophage subset to collagen. J Immunol. 2016;197:2229–2238. 27534550 10.4049/jimmunol.1501878

[19] Ohradanova‐Repic A , Machacek C , Fischer MB , Stockinger H . Differentiation of human monocytes and derived subsets of macrophages and dendritic cells by the HLDA10 monoclonal antibody panel. Clin Transl Immunol. 2016;5:e55. 10.1038/cti.2015.39 PMC4735061 26900469

[20] Cossarizza A , Chang HD , Radbruch A , et al. Guidelines for the use of flow cytometry and cell sorting in immunological studies. Eur J Immunol. 2017;47:1584. 29023707 10.1002/eji.201646632 PMC9165548

[21] Schiller HB , Szekeres A , Binder BR , Stockinger H , Leksa V . Mannose 6‐phosphate/insulin‐like growth factor 2 receptor limits cell invasion by controlling alphaVbeta3 integrin expression and proteolytic processing of urokinase‐type plasminogen activator receptor. Mol Biol Cell. 2009;20:745–756. 19037107 10.1091/mbc.E08-06-0569 PMC2633380

[22] Livak KJ , Schmittgen TD . Analysis of relative gene expression data using real‐time quantitative PCR and the 2(–Delta Delta C(T)) Method. Methods. 2001;25:402–408. 11846609 10.1006/meth.2001.1262

[23] Ohradanova‐Repic A , Machacek C , Charvet C , et al. Extracellular purine metabolism is the switchboard of immunosuppressive macrophages and a novel target to treat diseases with macrophage imbalances. Front Immunol. 2018;9:852. 29780382 10.3389/fimmu.2018.00852 PMC5946032

[24] Hattey E , Wojta J , Binder BR . Monoclonal antibodies against four different epitopes of plasminogen exhibit different effects on plasminogen activation kinetics. Thromb Haemost. 1987;58.

[25] Baqui AA , Meiller TF , Turng BF , Kelley JI . Functional changes in THP‐1 human monocytic cells after stimulation with lipopolysaccharide of oral microorganisms and granulocyte macrophage colony stimulating factor. Immunopharmacol Immunotoxicol. 1998;20:493–518. 9805230 10.3109/08923979809031512

[26] Elliott MR , Koster KM , Murphy PS . Efferocytosis signaling in the regulation of macrophage inflammatory responses. J Immunol. 2017;198:1387–1394. 28167649 10.4049/jimmunol.1601520 PMC5301545

[27] Birge RB , Boeltz S , Kumar S , et al. Phosphatidylserine is a global immunosuppressive signal in efferocytosis, infectious disease, and cancer. Cell Death Differ. 2016;23:962–978. 26915293 10.1038/cdd.2016.11 PMC4987730

[28] Gordon S , Pluddemann A . Macrophage clearance of apoptotic cells: a critical assessment. Front Immunol. 2018;9. 10.3389/fimmu.2018.00127 PMC5797608 29441073

[29] Schwende H , Fitzke E , Ambs P , Dieter P . Differences in the state of differentiation of THP‐1 cells induced by phorbol ester and 1,25‐dihydroxyvitamin D3. J Leukoc Biol. 1996;59:555–561. 8613704

[30] Hall SE , Savill JS , Henson PM , Haslett C . Apoptotic neutrophils are phagocytosed by fibroblasts with participation of the fibroblast vitronectin receptor and involvement of a mannose/fucose‐specific lectin. J Immunol. 1994;153:3218–3227. 7522254

[31] Ouyang L , Shi Z , Zhao S , et al. Programmed cell death pathways in cancer: a review of apoptosis, autophagy and programmed necrosis. Cell Prolif. 2012;45:487–498. 23030059 10.1111/j.1365-2184.2012.00845.x PMC6496669

[32] Mattson MP . Neuronal life‐and‐death signaling, apoptosis, and neurodegenerative disorders. Antioxid Redox Signal. 2006;8:1997–2006. 17034345 10.1089/ars.2006.8.1997

[33] Ravichandran KS . Beginnings of a good apoptotic meal: the find‐me and eat‐me signaling pathways. Immunity. 2011;35:445–455. 22035837 10.1016/j.immuni.2011.09.004 PMC3241945

[34] Kobayashi N , Karisola P , Pena‐Cruz V , et al. TIM‐1 and TIM‐4 glycoproteins bind phosphatidylserine and mediate uptake of apoptotic cells. Immunity. 2007;27:927–940. 18082433 10.1016/j.immuni.2007.11.011 PMC2757006

[35] DeKruyff RH , Bu X , Ballesteros A , et al. T cell/transmembrane, Ig, and mucin‐3 allelic variants differentially recognize phosphatidylserine and mediate phagocytosis of apoptotic cells. J Immunol. 2010;184:1918–1930. 20083673 10.4049/jimmunol.0903059 PMC3128800

[36] Park D , Tosello‐Trampont AC , Elliott MR , et al. BAI1 is an engulfment receptor for apoptotic cells upstream of the ELMO/Dock180/Rac module. Nature. 2007;450:430–434. 17960134 10.1038/nature06329

[37] Voss OH , Tian LJ , Murakami Y , Coligan JE , Krzewski K . Emerging role of CD300 receptors in regulating myeloid cell efferocytosis. Mol Cell Oncol. 2015;2. 10.4161/23723548.2014.964625 PMC4905414 27308512

[38] Park SY , Jung MY , Lee SJ , et al. Stabilin‐1 mediates phosphatidylserine‐dependent clearance of cell corpses in alternatively activated macrophages. J Cell Sci. 2009;122:3365–3373. 19726632 10.1242/jcs.049569

[39] Park SY , Jung MY , Kim HJ , et al. Rapid cell corpse clearance by stabilin‐2, a membrane phosphatidylserine receptor. Cell Death Differ. 2008;15:192–201. 17962816 10.1038/sj.cdd.4402242

[40] Hanayama R , Tanaka M , Miwa K , Shinohara A , Iwamatsu A , Nagata S . Identification of a factor that links apoptotic cells to phagocytes. Nature. 2002;417:182–187. 12000961 10.1038/417182a

[41] Jun JI , Kim KH , Lau LF . The matricellular protein CCN1 mediates neutrophil efferocytosis in cutaneous wound healing. Nat Commun. 2015;6. 10.1038/ncomms8386 PMC4480344 26077348

[42] Stepanek O , Brdicka T , Angelisova P , et al. Interaction of late apoptotic and necrotic cells with vitronectin. PLoS One. 2011;6:e19243. 21573223 10.1371/journal.pone.0019243 PMC3087723

[43] Seitz HM , Camenisch TD , Lemke G , Earp HS , Matsushima GK . Macrophages and dendritic cells use different Axl/Mertk/Tyro3 receptors in clearance of apoptotic cells. J Immunol. 2007;178:5635–5642. 17442946 10.4049/jimmunol.178.9.5635

[44] D'Mello V , Singh S , Wu Y , Birge RB . The urokinase plasminogen activator receptor promotes efferocytosis of apoptotic cells. J Biol Chem. 2009;284:17030–17038. 19383607 10.1074/jbc.M109.010066 PMC2719341

[45] Greenberg ME , Sun M , Zhang R , Febbraio M , Silverstein R , Hazen SL . Oxidized phosphatidylserine‐CD36 interactions play an essential role in macrophage‐dependent phagocytosis of apoptotic cells. J Exp Med. 2006;203:2613–2625. 17101731 10.1084/jem.20060370 PMC2118161

[46] Ogden CA , deCathehneau A , Hoffmann PR , et al. C1q and mannose binding lectin engagement of cell surface calreticulin and CD91 initiates macropinocytosis and uptake of apoptotic cells. J Exp Med. 2001;194:781–795. 11560994 10.1084/jem.194.6.781 PMC2195958

[47] Gregory CD , Devitt A , Moffatt O . Roles of ICAM‐3 and CD14 in the recognition and phagocytosis of apoptotic cells by macrophages. Biochem Soc Trans. 1998;26:644–649. 10047798 10.1042/bst0260644

[48] Hawkes C , Kabogo D , Amritraj A , Kar S . Up‐regulation of cation‐independent mannose 6‐phosphate receptor and endosomal‐lysosomal markers in surviving neurons after 192‐IgG‐saporin administrations into the adult rat brain. Am J Pathol. 2006;169:1140–1154. 17003474 10.2353/ajpath.2006.051208 PMC1698847

[49] Nykjaer A , Christensen EI , Vorum H , et al. Mannose 6‐phosphate/insulin‐like growth factor‐II receptor targets the urokinase receptor to lysosomes via a novel binding interaction. J Cell Biol. 1998;141:815–828. 9566979 10.1083/jcb.141.3.815 PMC2132758

[50] Amritraj A , Posse de Chaves EI , Hawkes C , Macdonald RG , Kar S . Single‐transmembrane domain IGF‐II/M6P receptor: potential interaction with G protein and its association with cholesterol‐rich membrane domains. Endocrinology. 2012;153:4784–4798. 22903618 10.1210/en.2012-1139

[51] Guzik K , Bzowska M , Smagur J , et al. A new insight into phagocytosis of apoptotic cells: proteolytic enzymes divert the recognition and clearance of polymorphonuclear leukocytes by macrophages. Cell Death Differ. 2007;14:171–182. 16628232 10.1038/sj.cdd.4401927

[52] Martin M , Leffler J , Blom AM . Annexin A2 and A5 serve as new ligands for C1q on apoptotic cells. J Biol Chem. 2012;287:33733–33744. 22879587 10.1074/jbc.M112.341339 PMC3460470

[53] Herren T , Swaisgood C , Plow EF . Regulation of plasminogen receptors. Front Biosci. 2003;8:d1–8. 12456318 10.2741/916

[54] Miles LA , Hawley SB , Baik N , Andronicos NM , Castellino FJ , Parmer RJ . Plasminogen receptors: the sine qua non of cell surface plasminogen activation. Front Biosci. 2005;10:1754–1762. 15769664 10.2741/1658

[55] Stillfried GE , Saunders DN , Ranson M . Plasminogen binding and activation at the breast cancer cell surface: the integral role of urokinase activity. Breast Cancer Res. 2007;9:R14. 17257442 10.1186/bcr1647 PMC1851380

[56] Andronicos NM , Chen EI , Baik N , et al. Proteomics‐based discovery of a novel, structurally unique, and developmentally regulated plasminogen receptor, Plg‐RKT, a major regulator of cell surface plasminogen activation. Blood;115:1319–1330. 10.1182/blood-2008-11-188938 PMC2826757 19897580

[57] Sugimoto MA , Ribeiro ALC , Costa BRC , et al. Plasmin and plasminogen induce macrophage reprogramming and regulate key steps of inflammation resolution via annexin A1. Blood. 2017;129:2896–2907. 28320709 10.1182/blood-2016-09-742825 PMC5445571

[58] Miles LA , Lighvani S , Baik N , et al. New insights into the role of Plg‐RKT in macrophage recruitment. Int Rev Cell Mol Biol. 2014;309:259–302. 24529725 10.1016/B978-0-12-800255-1.00005-3 PMC4060795

[59] Wu XW , Molinaro C , Johnson N , Casiano CA . Secondary necrosis is a source of proteolytically modified forms of specific intracellular autoantigens ‐ Implications for systemic autoimmunity. Arthritis Rheum. 2001;44:2642–2652. 11710720 10.1002/1529-0131(200111)44:11<2642::aid-art444>3.0.co;2-8

[60] Zwirzitz A , Reiter M , Skrabana R , et al. Lactoferrin is a natural inhibitor of plasminogen activation. J Biol Chem. 2018;293:8600–8613. 29669808 10.1074/jbc.RA118.003145 PMC5986228

[61] Lin SX , Mallet WG , Huang AY , Maxfield FR . Endocytosed cation‐independent mannose 6‐phosphate receptor traffics via the endocytic recycling compartment en route to the trans‐Golgi network and a subpopulation of late endosomes. Mol Biol Cell. 2004;15:721–733. 14595110 10.1091/mbc.E03-07-0497 PMC329388

[62] MacDonald RG , Pfeffer SR , Coussens L , et al. A single receptor binds both insulin‐like growth factor II and mannose‐6‐phosphate. Science. 1988;239:1134–1137. 2964083 10.1126/science.2964083

[63] Tong PY , Tollefsen SE , Kornfeld S . The cation‐independent mannose 6‐phosphate receptor binds insulin‐like growth factor II. J Biol Chem. 1988;263:2585–2588. 2963812

[64] Braulke T , Causin C , Waheed A , et al. Mannose 6‐phosphate/insulin‐like growth factor II receptor: distinct binding sites for mannose 6‐phosphate and insulin‐like growth factor II. Biochem Biophys Res Commun. 1988;150:1287–1293. 2963636 10.1016/0006-291x(88)90769-3

[65] Vreys V , Delande N , Zhang Z , et al. Cellular uptake of mammalian heparanase precursor involves low density lipoprotein receptor‐related proteins, mannose 6‐phosphate receptors, and heparan sulfate proteoglycans. J Biol Chem. 2005;280:33141–33148. 16046412 10.1074/jbc.M503007200

[66] Wood RJ , Hulett MD . Cell surface‐expressed cation‐independent mannose 6‐phosphate receptor (CD222) binds enzymatically active heparanase independently of mannose 6‐phosphate to promote extracellular matrix degradation. J Biol Chem. 2008;283:4165–4176. 18073203 10.1074/jbc.M708723200

[67] Blanchard F , Raher S , Duplomb L , et al. The mannose 6‐phosphate/insulin‐like growth factor II receptor is a nanomolar affinity receptor for glycosylated human leukemia inhibitory factor. J Biol Chem. 1998;273:20886–20893. 9694835 10.1074/jbc.273.33.20886

[68] Lee SJ , Nathans D . Proliferin secreted by cultured cells binds to mannose 6‐phosphate receptors. J Biol Chem. 1988;263:3521–3527. 2963825

[69] Volpert O , Jackson D , Bouck N , Linzer DI . The insulin‐like growth factor II/mannose 6‐phosphate receptor is required for proliferin‐induced angiogenesis. Endocrinology. 1996;137:3871–3876. 8756559 10.1210/endo.137.9.8756559

[70] Kovacina KS , Steele‐Perkins G , Purchio AF , et al. Interactions of recombinant and platelet transforming growth factor‐beta 1 precursor with the insulin‐like growth factor II/mannose 6‐phosphate receptor. Biochem Biophys Res Commun. 1989;160:393–403. 2540751 10.1016/0006-291x(89)91669-0

[71] Godar S , Horejsi V , Weidle UH , Binder BR , Hansmann C , Stockinger H . M6P/IGFII‐receptor complexes urokinase receptor and plasminogen for activation of transforming growth factor‐beta1. Eur J Immunol. 1999;29:1004–1013. 10092105 10.1002/(SICI)1521-4141(199903)29:03<1004::AID-IMMU1004>3.0.CO;2-Q

[72] Braulke T , Tippmer S , Neher E , von Figura K . Regulation of the mannose 6‐phosphate/IGF II receptor expression at the cell surface by mannose 6‐phosphate, insulin like growth factors and epidermal growth factor. EMBO J. 1989;8:681–686. 2542023 10.1002/j.1460-2075.1989.tb03426.x PMC400861

[73] van Meel E , Klumperman J . TGN exit of the cation‐independent mannose 6‐phosphate receptor does not require acid hydrolase binding. Cell Logist. 2014;4:e954441. 25610721 10.4161/21592780.2014.954441 PMC4292573

