# Supplementary figures and images for: The mannose 6‐phosphate/insulin‐like growth factor 2 receptor mediates plasminogen‐induced efferocytosis
Source: J Leukoc Biol. 2019 Jan 18;105(3):519–30. doi: 10.1002/JLB.1AB0417-160RR (PMC6392118; doi:10.1002/JLB.1AB0417-160RR)

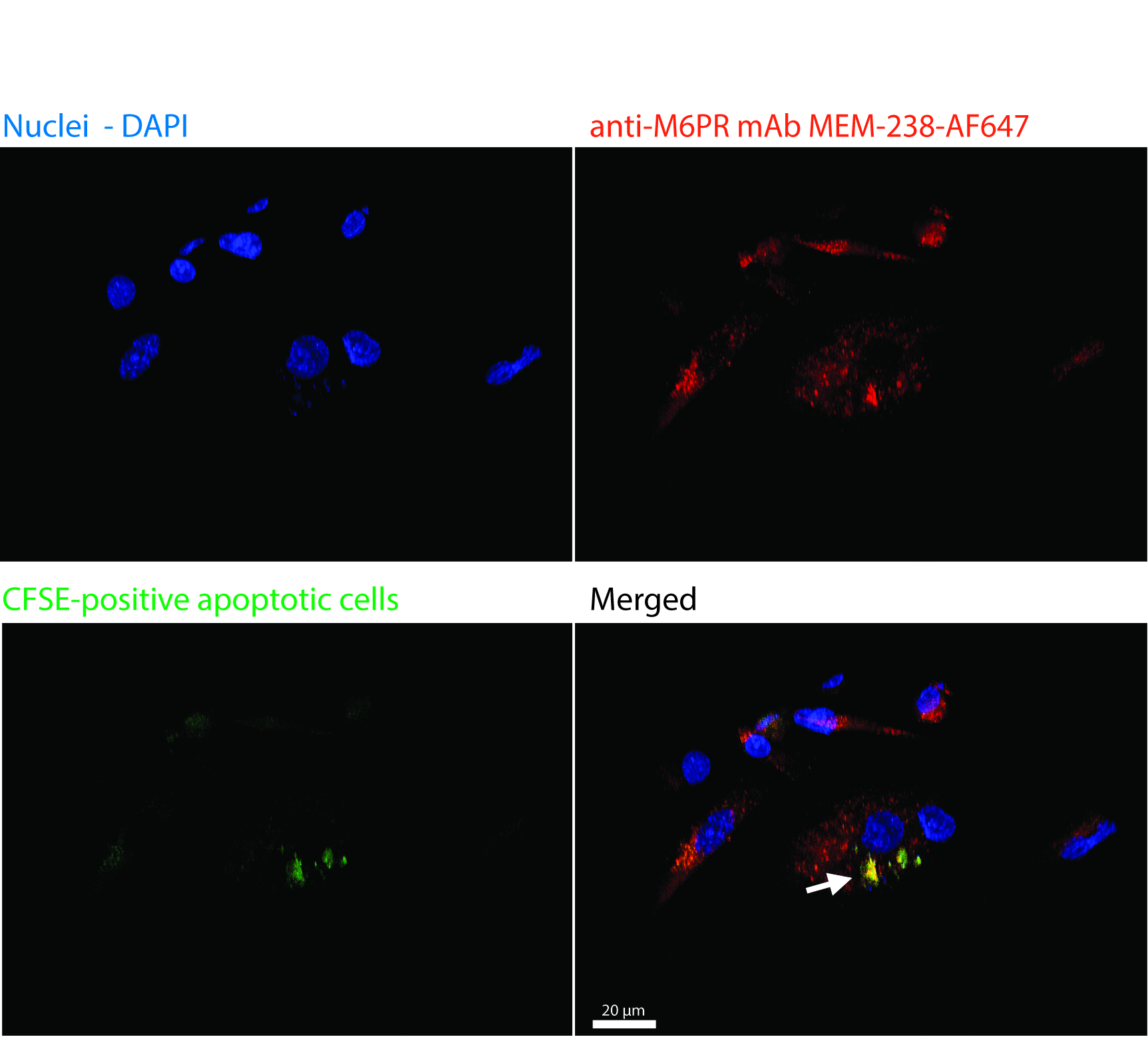

Supplement: Supplementary file 1 — Figure S1: Confocal microscopy analysis of Plg‐mediated efferocytosis by human macrophages. After incubation with CFSE‐labeled apoptotic Jurkat T cells (green) for 2 h, monocyte‐derived macrophages were fixed, permeabilized and stained with AF647‐conjugated anti‐M6P/IGF2R mAb MEM‐238 (red). Nuclei were stained with DAPI (blue). The slides were washed and analyzed by confocal microscopy. A 3D confocal image reconstructed from 15 z‐stacks in the total range of 4.18 μm is shown. Arrows point to the region of colocalization of M6P/IGF2R and apoptotic bodies; the scale represents 20 μm. [file JLB-105-519-s001.tif]
